# Tetraaqua­tetrakis­{μ_2_-[1-(carb­oxylato­meth­yl)cyclo­hex­yl]methanaminium}bis(μ_3_-hydroxido)bis(nitrato-κ^2^ *O*,*O*′)tetrazinc(II)

**DOI:** 10.1107/S1600536811011020

**Published:** 2011-04-13

**Authors:** Elise J. C. de Vries, Caryn Gamble, Ahmed Shaikjee

**Affiliations:** a Molecular Science Institute, School of Chemistry, University of the Witwatersrand, PO WITS, 2050, Johannesburg, South Africa

## Abstract

As the title gabapentin complex, [Zn_4_(OH)_2_(NO_3_)_2_(C_9_H_17_NO_2_)_4_(H_2_O)_4_](NO_3_)_4_ is located about a centre of inversion, the asymmetric unit contains two disordered nitrate ions and half a complex mol­ecule. The two zinc ions have different coordination environments: one is slightly distorted octa­hedral and the other is trigonal–pyramidal. The conformation of the gabapentin mol­ecule is defined by the formation of two intra­molecular O—H⋯O hydrogen bonds. Furthermore, the ammonium H atoms are involved in numerous hydrogen bonds with the disordered nitrate anions.

## Related literature

For related transition metal complexes with gabapentin, see: Braga *et al.* (2008 ▶). For structures with hexa- and tetra-coordinated zinc atoms, see: Clegg *et al.* (1991 ▶); Karmakar & Baruah (2008 ▶). For the structure of a gabapentin nitrate salt, see: de Vries *et al.* (2011 ▶).
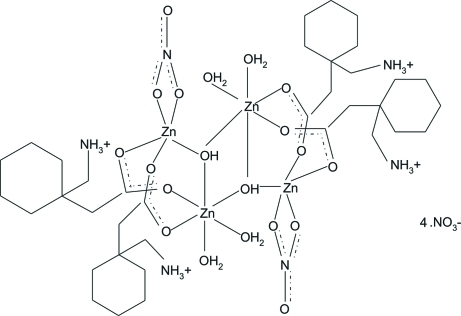


## Experimental

### 

#### Crystal data

[Zn_4_(OH)_2_(NO_3_)_2_(C_9_H_17_NO_2_)_4_(H_2_O)_4_](NO_3_)_4_
*M* *_r_* = 1424.64
Triclinic, 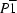

*a* = 10.0160 (2) Å
*b* = 11.3524 (2) Å
*c* = 14.2480 (2) Å
α = 88.740 (1)°
β = 74.021 (1)°
γ = 67.295 (1)°
*V* = 1430.28 (4) Å^3^
*Z* = 1
Mo *K*α radiation
μ = 1.76 mm^−1^
*T* = 173 K
0.51 × 0.30 × 0.22 mm

#### Data collection

Bruker APEXII CCD diffractometer
Absorption correction: multi-scan (*SADABS*; Bruker, 2005 ▶) *T*_min_ = 0.468, *T*_max_ = 0.698
21157 measured reflections
6238 independent reflections
5401 reflections with *I* > 2σ(*I*)
*R*_int_ = 0.027

#### Refinement

*R*[*F* ^2^ > 2σ(*F* ^2^)] = 0.047
*wR*(*F* ^2^) = 0.128
*S* = 1.05
6238 reflections
363 parameters
6 restraints
H-atom parameters constrained
Δρ_max_ = 1.54 e Å^−3^
Δρ_min_ = −1.42 e Å^−3^

### 

Data collection: *APEX2* (Bruker, 2005 ▶); cell refinement: *SAINT-NT* (Bruker, 2005 ▶); data reduction: *SAINT-NT*; program(s) used to solve structure: *SHELXS97* (Sheldrick, 2008) ▶; program(s) used to refine structure: *SHELXL97* (Sheldrick, 2008) ▶; molecular graphics: *X-SEED* (Barbour, 2001 ▶; Atwood & Barbour, 2003 ▶); software used to prepare material for publication: *X-SEED*.

## Supplementary Material

Crystal structure: contains datablocks global, I. DOI: 10.1107/S1600536811011020/ez2233sup1.cif

Structure factors: contains datablocks I. DOI: 10.1107/S1600536811011020/ez2233Isup2.hkl

Additional supplementary materials:  crystallographic information; 3D view; checkCIF report

## Figures and Tables

**Table 1:** Selected bond lengths (Å)

Zn1—O1	2.075 (2)
Zn1—O5^i^	2.075 (2)
Zn1—O5	2.077 (2)
Zn1—O6	2.099 (2)
Zn1—O7	2.109 (3)
Zn1—O3	2.127 (2)
Zn2—O4	1.933 (3)
Zn2—O5	1.954 (2)
Zn2—O2	1.979 (3)
Zn2—O2*E*	1.979 (7)
Zn2—O3*B*	2.122 (5)

Symmetry code: (i) 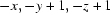.

**Table 2:** Hydrogen-bond geometry (Å, °)

*D*—H⋯*A*	*D*—H	H⋯*A*	*D*⋯*A*	*D*—H⋯*A*
N1—H1*C*⋯O1	0.91	2.23	2.918 (4)	132
N1—H1*D*⋯O2*C*^ii^	0.91	2.18	3.003 (9)	149
N1—H1*E*⋯O1*B*^ii^	0.91	2.57	3.015 (6)	111
N1—H1*E*⋯O2*D*^iii^	0.91	2.25	3.090 (8)	153
N1—H1*E*⋯O3*D*^iii^	0.91	2.31	3.037 (8)	136
N2—H2*C*⋯O1*D*^iv^	0.91	2.00	2.906 (8)	172
N2—H2*D*⋯O1*C*^ii^	0.91	2.02	2.884 (9)	158
N2—H2*E*⋯O3	0.91	2.01	2.748 (5)	138
O5—H5*C*⋯O1*D*	0.95	1.86	2.809 (8)	173
O6—H6*C*⋯O2*C*^ii^	0.98	1.97	2.944 (7)	170
O6—H6*C*⋯O2*C*^i^	0.98	2.01	2.819 (7)	138
O6—H6*D*⋯O3*D*^iv^	0.95	2.58	3.010 (9)	108
O6—H6*D*⋯O3*B*^i^	0.95	2.00	2.927 (5)	163
O7—H7*C*⋯O3*C*^i^	0.95	1.78	2.735 (9)	178
O7—H7*D*⋯O2*D*	0.96	1.87	2.819 (8)	173

Symmetry codes: (i) 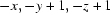; (ii) 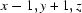; (iii) 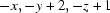; (iv) 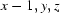.

**Fig. 1.**
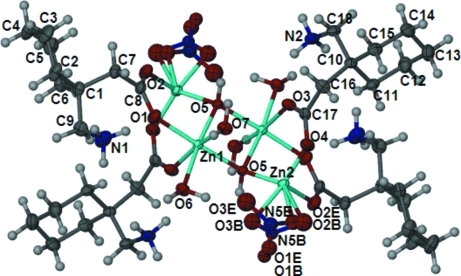
The atomic numbering scheme of complex (I). Uncoordinated anions are not shown. Displacement ellipsoids are drawn at 50% probability level.

**Fig. 2.**
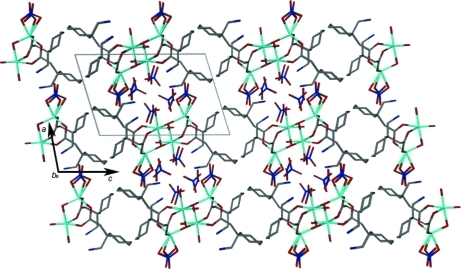
Packing diagram of complex (I) viewed down the b axis. Hydrogen atoms omitted for clarity.

## supplementary crystallographic information

### Comment

The first transition metal complexes of gabapentin were published by Braga (Braga *et al.*, 2008). Complexes were obtained by grinding together gabapentin and the inorganic salts ZnCl_2_ and CuCl_2_.2H_2_O. In the resulting crystals of the zinc complex the Zn(II) cation is tetrahedrally coordinated to two chloride anions and two zwitterionic gabapentin molecules. In this study we investigated the effect of the nitrate counterion on the nature of the metal gabapentin complex formed.

The asymmetric unit contains [Zn_2_(Gpn)_2_(H_2_O)_2_(NO_3_)OH]^2+^.(NO_3_^-^)_2_ (complex (I)). This molecule is situated around the centre of inversion (0, 1/2, 1/2). The two zinc metal ions in the asymmetric unit have different coordination environments. The first zinc ion (Zn1) is in a slightly distorted octahedral environment, coordinated to two water oxygen atoms, two oxygen hydroxyl atoms (one generated by symmetry) and two gabapentin carboxylate oxygen atoms. The water and hydroxyl oxygen atoms are in both the axial and equatorial positions. The second zinc ion (Zn2) is in a trigonal pyramidal coordination environment and is coordinated to two gabapentin carboxylate oxygen atoms, one hydroxyl oxygen atom and an oxygen atom of a disordered nitrate molecule (found in the axial position). The two metal ions are therefore linked by the two hydroxyl groups and two gabapentins which act as a bridge (Fig. 1). As the asymmetric unit is located around a centre of inversion each hydroxyl group is bonded to three zinc metal ions, by symmetry. This type of hexa and tetra coordinated zinc has been observed before (Clegg *et al.*, 1991; Karmakar & Baruah, 2008). Each gabapentin is in the chair conformation, with the ammonium group in the equatorial position. The conformation of gabapentin can be described by torsion angles which indicate that the two molecules are quite similar (Table 1). Each of the nitrate counterions was found to be disordered over two positions within the structure, with one of the nitrate counterions coordinating to a metal centre.

The crystal packing is determined by hydrogen bonds involving the nitrate anions, carboxylic acid and NH_3_^+^ groups. The conformation of the gabapentin molecule is defined by the formation of two intramolecular hydrogen bonds between the ammonium and carboxylic acid oxygen atoms of gabapentin. This bond plays an important role in the determination of the structural properties of the metal complex and is also observed in Braga's Zn(II) complex (Braga *et al.*, 2008). Furthermore, the two NH_3_^+^ groups are involved in numerous hydrogen bonds, some of which are bifurcated, with the three disordered nitrate anions. The molecular conformation is further stabilized by four intermolecular C—H···O bonds which are formed between gabapentin and the nitrate anions with distances and angles in the range of 3.138–3.394 Å and 126–164 ° respectively.

### Experimental

Gabapentin was purchased from Sigma-Aldrich. Complex (I) is obtained by reflux solution crystallization. The metal salt (0.371 g) and gabapentin (0.428 g) were combined in 1:2 stoichiometric proportions, then dissolved in 25 ml distilled water. The solution was refluxed at 60 degrees Celcius for an hour, then allowed to cool in a fridge at 12 degrees Celcius (until crystals formed).

### Refinement

All H atoms were positioned geometrically and allowed to ride on their respective parent atoms, with C—H bond lengths of 0.99 (aromatic CH) 1.00 (methine CH), 0.99 (methylene CH_2_) and 0.98 Å (methyl CH_3_), and with *U*_iso_(H) = 1.2 or 1.5 times *U*_eq_(C). The nitrate anions are disordered over two positions and their occupancies were refined freely, with final occupancies of 0.537 (7), 0.548 (7) and 0.513 (10) for the nitrates labelled B, C and D respectively. Geometric constraints were placed on some of the nitrate anions to improve their geometries and thermal ellipsoid parameters. The *hkl* reflection 521 was omitted from the refinement as I(obs) and I(calc) differed more than 10 times σω.

### Crystal data

**Table:**

[Zn_4_(OH)_2_(NO_3_)_2_(C_9_H_17_NO_2_)_4_(H_2_O)_4_](NO_3_)_4_	*Z* = 1
*M**_r_* = 1424.64	*F*(000) = 740
Triclinic, *P*1	*D*_x_ = 1.654 Mg m^−^^3^
Hall symbol: -P 1	Mo *K*α radiation, λ = 0.71073 Å
*a* = 10.0160 (2) Å	Cell parameters from 9598 reflections
*b* = 11.3524 (2) Å	θ = 2.3–28.3°
*c* = 14.2480 (2) Å	µ = 1.76 mm^−^^1^
α = 88.740 (1)°	*T* = 173 K
β = 74.021 (1)°	Plate, colourless
γ = 67.295 (1)°	0.51 × 0.30 × 0.22 mm
*V* = 1430.28 (4) Å^3^	

### Data collection

**Table:**

Bruker APEXII CCD diffractometer	6238 independent reflections
Radiation source: fine-focus sealed tube	5401 reflections with *I* > 2σ(*I*)
graphite	*R*_int_ = 0.027
φ and ω scans	θ_max_ = 27.0°, θ_min_ = 1.5°
Absorption correction: multi-scan (*SADABS*; Bruker, 2005)	*h* = −12→12
*T*_min_ = 0.468, *T*_max_ = 0.698	*k* = −14→14
21157 measured reflections	*l* = −18→18

### Refinement

**Table:**

Refinement on *F*^2^	Primary atom site location: structure-invariant direct methods
Least-squares matrix: full	Secondary atom site location: difference Fourier map
*R*[*F*^2^ > 2σ(*F*^2^)] = 0.047	Hydrogen site location: inferred from neighbouring sites
*wR*(*F*^2^) = 0.128	H-atom parameters constrained
*S* = 1.05	*w* = 1/[σ^2^(*F*_o_^2^) + (0.0613*P*)^2^ + 3.8991*P*] where *P* = (*F*_o_^2^ + 2*F*_c_^2^)/3
6238 reflections	(Δ/σ)_max_ = 0.013
363 parameters	Δρ_max_ = 1.54 e Å^−^^3^
6 restraints	Δρ_min_ = −1.42 e Å^−^^3^

### Special details

**Table:**

Experimental. Absorption corrections were made using the program *SADABS* (Sheldrick, 2004)
Geometry. All e.s.d.'s (except the e.s.d. in the dihedral angle between two l.s. planes) are estimated using the full covariance matrix. The cell e.s.d.'s are taken into account individually in the estimation of e.s.d.'s in distances, angles and torsion angles; correlations between e.s.d.'s in cell parameters are only used when they are defined by crystal symmetry. An approximate (isotropic) treatment of cell e.s.d.'s is used for estimating e.s.d.'s involving l.s. planes.
Refinement. Refinement of *F*^2^ against ALL reflections. The weighted *R*-factor *wR* and goodness of fit *S* are based on *F*^2^, conventional *R*-factors *R* are based on *F*, with *F* set to zero for negative *F*^2^. The threshold expression of *F*^2^ > σ(*F*^2^) is used only for calculating *R*-factors(gt) *etc*. and is not relevant to the choice of reflections for refinement. *R*-factors based on *F*^2^ are statistically about twice as large as those based on *F*, and *R*- factors based on ALL data will be even larger.

### Fractional atomic coordinates and isotropic or equivalent isotropic displacement parameters (Å^2^)

**Table:**

	*x*	*y*	*z*	*U*_iso_*/*U*_eq_	Occ. (<1)
Zn1	−0.08278 (4)	0.62842 (3)	0.46097 (3)	0.01972 (11)	
O1	−0.0166 (3)	0.7519 (2)	0.3682 (2)	0.0336 (6)	
N1	−0.2171 (4)	1.0031 (3)	0.3320 (3)	0.0435 (8)	
H1D	−0.3130	1.0069	0.3452	0.065*	
H1E	−0.2199	1.0839	0.3362	0.065*	
H1C	−0.1728	0.9571	0.3763	0.065*	
C1	0.0355 (4)	0.9276 (3)	0.2009 (3)	0.0281 (7)	
Zn2	0.22789 (4)	0.47355 (4)	0.30465 (3)	0.02482 (12)	
H6D	−0.3494	0.7287	0.5512	0.15 (4)*	
H7C	−0.1625	0.8249	0.5891	0.058 (15)*	
H7D	−0.0066	0.7513	0.5868	0.08 (2)*	
H5C	0.1996	0.5382	0.4626	0.062 (16)*	
H6C	−0.3895	0.8254	0.4791	0.13 (3)*	
O2	0.2232 (3)	0.6464 (2)	0.2767 (2)	0.0358 (6)	
N2	−0.4080 (3)	0.6146 (3)	0.3453 (2)	0.0302 (6)	
H2C	−0.4994	0.6262	0.3879	0.045*	
H2D	−0.4041	0.6923	0.3327	0.045*	
H2E	−0.3333	0.5686	0.3722	0.045*	
C2	0.0420 (5)	1.0591 (3)	0.1794 (3)	0.0394 (9)	
H2A	−0.0225	1.0997	0.1364	0.047*	
H2B	−0.0004	1.1148	0.2418	0.047*	
O3	−0.1127 (3)	0.5294 (2)	0.34852 (18)	0.0287 (5)	
C3	0.2008 (6)	1.0517 (4)	0.1306 (4)	0.0465 (11)	
H3A	0.2631	1.0204	0.1762	0.056*	
H3B	0.1962	1.1385	0.1156	0.056*	
O4	0.1073 (3)	0.4364 (3)	0.23377 (19)	0.0341 (6)	
C4	0.2743 (6)	0.9621 (4)	0.0362 (3)	0.0498 (11)	
H4A	0.3799	0.9542	0.0083	0.060*	
H4B	0.2189	0.9987	−0.0124	0.060*	
O5	0.1376 (2)	0.5004 (2)	0.44675 (17)	0.0212 (4)	
C5	0.2734 (5)	0.8296 (4)	0.0554 (3)	0.0411 (9)	
H5A	0.3152	0.7751	−0.0076	0.049*	
H5B	0.3391	0.7888	0.0976	0.049*	
O6	−0.3136 (3)	0.7413 (2)	0.48426 (19)	0.0302 (5)	
C6	0.1145 (5)	0.8373 (4)	0.1050 (3)	0.0334 (8)	
H6A	0.1199	0.7502	0.1198	0.040*	
H6B	0.0527	0.8677	0.0590	0.040*	
O7	−0.0932 (3)	0.7376 (2)	0.5826 (2)	0.0321 (6)	
C7	0.1148 (4)	0.8735 (3)	0.2808 (3)	0.0295 (7)	
H7A	0.2222	0.8614	0.2559	0.035*	
H7B	0.0671	0.9370	0.3391	0.035*	
C8	0.1070 (4)	0.7472 (3)	0.3114 (3)	0.0278 (7)	
C9	−0.1283 (4)	0.9397 (3)	0.2312 (3)	0.0353 (8)	
H9A	−0.1791	0.9893	0.1840	0.042*	
H9B	−0.1286	0.8529	0.2270	0.042*	
C10	−0.2366 (4)	0.5177 (3)	0.1742 (2)	0.0237 (6)	
C11	−0.2081 (4)	0.6402 (3)	0.1505 (3)	0.0290 (7)	
H11A	−0.2157	0.6844	0.2121	0.035*	
H11B	−0.1040	0.6161	0.1069	0.035*	
C12	−0.3194 (5)	0.7331 (4)	0.1010 (3)	0.0396 (9)	
H12A	−0.4226	0.7653	0.1473	0.048*	
H12B	−0.2919	0.8075	0.0842	0.048*	
C13	−0.3179 (5)	0.6677 (4)	0.0082 (3)	0.0435 (10)	
H13A	−0.3944	0.7285	−0.0203	0.052*	
H13B	−0.2175	0.6432	−0.0406	0.052*	
C14	−0.3516 (5)	0.5490 (4)	0.0299 (3)	0.0389 (9)	
H14A	−0.3427	0.5051	−0.0322	0.047*	
H14B	−0.4568	0.5746	0.0724	0.047*	
C15	−0.2431 (4)	0.4568 (4)	0.0807 (3)	0.0326 (8)	
H15A	−0.1404	0.4218	0.0339	0.039*	
H15B	−0.2736	0.3841	0.0982	0.039*	
C16	−0.1039 (4)	0.4168 (3)	0.2054 (3)	0.0312 (8)	
H16A	−0.1408	0.3548	0.2418	0.037*	
H16B	−0.0240	0.3689	0.1451	0.037*	
C17	−0.0319 (4)	0.4651 (3)	0.2677 (2)	0.0256 (7)	
C18	−0.3875 (4)	0.5441 (4)	0.2524 (3)	0.0297 (7)	
H18A	−0.4709	0.5941	0.2248	0.036*	
H18B	−0.3957	0.4611	0.2675	0.036*	
O1A	0.3312 (7)	0.6003 (6)	0.5027 (5)	0.0231 (14)*	0.487 (10)
O2A	0.1479 (8)	0.7922 (7)	0.5821 (6)	0.0357 (16)*	0.487 (10)
O3A	0.3675 (8)	0.7772 (7)	0.5194 (5)	0.053 (2)*	0.487 (10)
N3A	0.2773 (8)	0.7269 (8)	0.5350 (6)	0.0244 (17)*	0.487 (10)
O1D	0.3120 (7)	0.6272 (7)	0.4840 (5)	0.0316 (16)*	0.513 (10)
O2D	0.1653 (7)	0.7624 (6)	0.6064 (5)	0.0304 (14)*	0.513 (10)
O3D	0.3889 (8)	0.7207 (7)	0.5785 (6)	0.060 (2)*	0.513 (10)
N3D	0.2932 (8)	0.7002 (7)	0.5536 (6)	0.0251 (17)*	0.513 (10)
O1B	0.6378 (5)	0.2233 (5)	0.2221 (4)	0.0333 (13)*	0.537 (7)
O2B	0.4562 (6)	0.3503 (6)	0.1739 (4)	0.0597 (19)*	0.537 (7)
O3B	0.4324 (6)	0.3401 (6)	0.3257 (3)	0.0548 (19)*	0.537 (7)
N5B	0.5111 (8)	0.2971 (7)	0.2415 (5)	0.0284 (15)*	0.537 (7)
O1E	0.6232 (7)	0.1961 (7)	0.1990 (6)	0.0391 (17)*	0.463 (7)
O2E	0.4354 (7)	0.3790 (6)	0.2182 (5)	0.0358 (16)*	0.463 (7)
O3E	0.4128 (8)	0.2302 (7)	0.3050 (6)	0.052 (2)*	0.463 (7)
N5E	0.4917 (9)	0.2665 (9)	0.2375 (6)	0.0333 (19)*	0.463 (7)
O1C	0.5177 (11)	−0.1170 (9)	0.3201 (7)	0.072 (2)*	0.548 (7)
O2C	0.4870 (8)	−0.0027 (6)	0.4501 (5)	0.0575 (19)*	0.548 (7)
O3C	0.2965 (9)	0.0137 (9)	0.3980 (6)	0.072 (2)*	0.548 (7)
N4C	0.4311 (8)	−0.0360 (6)	0.3883 (6)	0.0334 (15)*	0.548 (7)
O1F	0.5650 (7)	−0.1201 (6)	0.3312 (5)	0.0359 (16)*	0.452 (7)
O2F	0.3302 (8)	−0.0551 (10)	0.4016 (8)	0.090*	0.452 (7)
O3F	0.4270 (10)	0.0782 (5)	0.3488 (8)	0.090*	0.452 (7)
N4F	0.4427 (11)	−0.0271 (10)	0.3500 (9)	0.048 (2)*	0.452 (7)

### Atomic displacement parameters (Å^2^)

**Table:**

	*U*^11^	*U*^22^	*U*^33^	*U*^12^	*U*^13^	*U*^23^
Zn1	0.01629 (18)	0.01734 (18)	0.0228 (2)	−0.00489 (14)	−0.00405 (14)	0.00329 (13)
O1	0.0291 (13)	0.0243 (12)	0.0391 (15)	−0.0072 (10)	−0.0029 (11)	0.0110 (11)
N1	0.0398 (19)	0.0292 (17)	0.046 (2)	−0.0079 (15)	0.0030 (16)	−0.0007 (15)
C1	0.0355 (19)	0.0163 (15)	0.0281 (17)	−0.0071 (14)	−0.0070 (15)	0.0030 (13)
Zn2	0.01772 (19)	0.0238 (2)	0.0265 (2)	−0.00436 (15)	−0.00230 (15)	0.00454 (15)
O2	0.0307 (13)	0.0254 (13)	0.0400 (15)	−0.0058 (11)	−0.0013 (11)	0.0105 (11)
N2	0.0205 (14)	0.0341 (16)	0.0308 (16)	−0.0091 (12)	−0.0016 (12)	−0.0033 (12)
C2	0.053 (2)	0.0182 (16)	0.036 (2)	−0.0098 (16)	−0.0021 (18)	0.0067 (14)
O3	0.0200 (11)	0.0299 (13)	0.0305 (13)	−0.0043 (10)	−0.0061 (10)	−0.0058 (10)
C3	0.060 (3)	0.0240 (19)	0.051 (3)	−0.0201 (19)	−0.003 (2)	0.0099 (17)
O4	0.0217 (12)	0.0445 (15)	0.0274 (13)	−0.0060 (11)	−0.0035 (10)	−0.0036 (11)
C4	0.055 (3)	0.043 (2)	0.039 (2)	−0.018 (2)	0.002 (2)	0.0120 (19)
O5	0.0187 (10)	0.0199 (10)	0.0265 (12)	−0.0089 (9)	−0.0071 (9)	0.0052 (9)
C5	0.046 (2)	0.033 (2)	0.032 (2)	−0.0099 (18)	0.0000 (17)	−0.0019 (16)
O6	0.0212 (12)	0.0278 (13)	0.0323 (14)	−0.0002 (10)	−0.0071 (10)	−0.0027 (10)
C6	0.040 (2)	0.0271 (17)	0.0289 (18)	−0.0081 (15)	−0.0104 (16)	−0.0003 (14)
O7	0.0291 (13)	0.0258 (12)	0.0380 (14)	−0.0072 (10)	−0.0093 (11)	−0.0040 (10)
C7	0.0379 (19)	0.0247 (16)	0.0286 (18)	−0.0152 (15)	−0.0099 (15)	0.0066 (13)
C8	0.0304 (18)	0.0249 (16)	0.0272 (17)	−0.0104 (14)	−0.0080 (14)	0.0087 (13)
C9	0.0336 (19)	0.0225 (17)	0.042 (2)	−0.0030 (15)	−0.0105 (17)	0.0019 (15)
C10	0.0219 (15)	0.0231 (15)	0.0271 (16)	−0.0104 (13)	−0.0058 (13)	−0.0022 (12)
C11	0.0295 (17)	0.0307 (18)	0.0304 (18)	−0.0179 (15)	−0.0054 (14)	0.0019 (14)
C12	0.051 (2)	0.033 (2)	0.040 (2)	−0.0197 (18)	−0.0175 (19)	0.0102 (16)
C13	0.051 (3)	0.050 (2)	0.032 (2)	−0.022 (2)	−0.0153 (19)	0.0107 (18)
C14	0.041 (2)	0.049 (2)	0.034 (2)	−0.0224 (19)	−0.0156 (17)	0.0004 (17)
C15	0.0343 (19)	0.0344 (19)	0.0306 (18)	−0.0160 (16)	−0.0073 (15)	−0.0064 (15)
C16	0.0285 (18)	0.0251 (17)	0.0325 (19)	−0.0025 (14)	−0.0084 (15)	−0.0083 (14)
C17	0.0217 (16)	0.0234 (16)	0.0250 (16)	−0.0023 (13)	−0.0060 (13)	0.0014 (13)
C18	0.0262 (17)	0.0346 (18)	0.0312 (18)	−0.0165 (15)	−0.0056 (14)	−0.0015 (14)

### Geometric parameters (Å, °)

**Table:**

Zn1—O1	2.075 (2)	O7—H7C	0.9540
Zn1—O5^i^	2.075 (2)	O7—H7D	0.9559
Zn1—O5	2.077 (2)	C7—C8	1.513 (5)
Zn1—O6	2.099 (2)	C7—H7A	0.9900
Zn1—O7	2.109 (3)	C7—H7B	0.9900
Zn1—O3	2.127 (2)	C9—H9A	0.9900
Zn1—Zn1^i^	3.1029 (7)	C9—H9B	0.9900
Zn1—Zn2	3.1412 (5)	C10—C18	1.534 (5)
O1—C8	1.262 (4)	C10—C11	1.537 (5)
N1—C9	1.490 (5)	C10—C15	1.542 (5)
N1—H1D	0.9100	C10—C16	1.550 (5)
N1—H1E	0.9100	C11—C12	1.531 (5)
N1—H1C	0.9100	C11—H11A	0.9900
C1—C9	1.528 (5)	C11—H11B	0.9900
C1—C2	1.540 (5)	C12—C13	1.525 (6)
C1—C6	1.544 (5)	C12—H12A	0.9900
C1—C7	1.548 (5)	C12—H12B	0.9900
Zn2—O4	1.933 (3)	C13—C14	1.516 (6)
Zn2—O5	1.954 (2)	C13—H13A	0.9900
Zn2—O2	1.979 (3)	C13—H13B	0.9900
Zn2—O2E	1.979 (7)	C14—C15	1.524 (6)
Zn2—O3B	2.122 (5)	C14—H14A	0.9900
Zn2—O2B	2.440 (6)	C14—H14B	0.9900
O2—C8	1.260 (4)	C15—H15A	0.9900
N2—C18	1.485 (4)	C15—H15B	0.9900
N2—H2C	0.9100	C16—C17	1.513 (5)
N2—H2D	0.9100	C16—H16A	0.9900
N2—H2E	0.9100	C16—H16B	0.9900
C2—C3	1.520 (6)	C18—H18A	0.9900
C2—H2A	0.9900	C18—H18B	0.9900
C2—H2B	0.9900	O1A—N3A	1.365 (10)
O3—C17	1.258 (4)	O2A—N3A	1.217 (10)
C3—C4	1.524 (6)	O3A—N3A	1.214 (10)
C3—H3A	0.9900	O1D—N3D	1.233 (9)
C3—H3B	0.9900	O2D—N3D	1.224 (9)
O4—C17	1.253 (4)	O3D—N3D	1.210 (10)
C4—C5	1.526 (6)	O1B—N5B	1.177 (7)
C4—H4A	0.9900	O2B—N5B	1.266 (7)
C4—H4B	0.9900	O3B—N5B	1.228 (7)
O5—Zn1^i^	2.075 (2)	O1E—N5E	1.218 (10)
O5—H5C	0.9537	O2E—N5E	1.240 (11)
C5—C6	1.522 (6)	O3E—N5E	1.239 (11)
C5—H5A	0.9900	O1C—N4C	1.225 (11)
C5—H5B	0.9900	O2C—N4C	1.297 (9)
O6—H6D	0.9503	O3C—N4C	1.212 (10)
O6—H6C	0.9812	O1F—N4F	1.233 (12)
C6—H6A	0.9900	O2F—N4F	1.312 (12)
C6—H6B	0.9900	O3F—N4F	1.144 (12)
			
O1—Zn1—O5^i^	177.11 (9)	H5A—C5—H5B	108.0
O1—Zn1—O5	93.85 (9)	Zn1—O6—H6D	98.6
O5^i^—Zn1—O5	83.28 (9)	Zn1—O6—H6C	146.2
O1—Zn1—O6	92.92 (10)	H6D—O6—H6C	102.2
O5^i^—Zn1—O6	89.95 (10)	C5—C6—C1	113.2 (3)
O5—Zn1—O6	173.15 (10)	C5—C6—H6A	108.9
O1—Zn1—O7	89.88 (11)	C1—C6—H6A	108.9
O5^i^—Zn1—O7	89.98 (10)	C5—C6—H6B	108.9
O5—Zn1—O7	93.55 (9)	C1—C6—H6B	108.9
O6—Zn1—O7	87.50 (10)	H6A—C6—H6B	107.8
O1—Zn1—O3	93.96 (11)	Zn1—O7—H7C	113.3
O5^i^—Zn1—O3	86.62 (10)	Zn1—O7—H7D	120.8
O5—Zn1—O3	95.10 (9)	H7C—O7—H7D	98.9
O6—Zn1—O3	83.38 (9)	C8—C7—C1	113.2 (3)
O7—Zn1—O3	170.27 (10)	C8—C7—H7A	108.9
O1—Zn1—Zn1^i^	135.46 (7)	C1—C7—H7A	108.9
O5^i^—Zn1—Zn1^i^	41.67 (6)	C8—C7—H7B	108.9
O5—Zn1—Zn1^i^	41.61 (6)	C1—C7—H7B	108.9
O6—Zn1—Zn1^i^	131.61 (8)	H7A—C7—H7B	107.8
O7—Zn1—Zn1^i^	92.37 (7)	O2—C8—O1	125.5 (3)
O3—Zn1—Zn1^i^	91.15 (7)	O2—C8—C7	117.5 (3)
O1—Zn1—Zn2	70.09 (7)	O1—C8—C7	117.0 (3)
O5^i^—Zn1—Zn2	107.55 (6)	N1—C9—C1	114.2 (3)
O5—Zn1—Zn2	37.41 (6)	N1—C9—H9A	108.7
O6—Zn1—Zn2	145.77 (7)	C1—C9—H9A	108.7
O7—Zn1—Zn2	120.73 (7)	N1—C9—H9B	108.7
O3—Zn1—Zn2	69.00 (6)	C1—C9—H9B	108.7
Zn1^i^—Zn1—Zn2	70.725 (14)	H9A—C9—H9B	107.6
C8—O1—Zn1	135.0 (2)	C18—C10—C11	112.7 (3)
C9—N1—H1D	109.5	C18—C10—C15	106.9 (3)
C9—N1—H1E	109.5	C11—C10—C15	109.1 (3)
H1D—N1—H1E	109.5	C18—C10—C16	110.6 (3)
C9—N1—H1C	109.5	C11—C10—C16	110.7 (3)
H1D—N1—H1C	109.5	C15—C10—C16	106.5 (3)
H1E—N1—H1C	109.5	C12—C11—C10	113.1 (3)
C9—C1—C2	110.3 (3)	C12—C11—H11A	109.0
C9—C1—C6	105.8 (3)	C10—C11—H11A	109.0
C2—C1—C6	108.6 (3)	C12—C11—H11B	109.0
C9—C1—C7	112.0 (3)	C10—C11—H11B	109.0
C2—C1—C7	109.0 (3)	H11A—C11—H11B	107.8
C6—C1—C7	111.0 (3)	C13—C12—C11	111.1 (3)
O4—Zn2—O5	113.77 (10)	C13—C12—H12A	109.4
O4—Zn2—O2	107.78 (12)	C11—C12—H12A	109.4
O5—Zn2—O2	100.42 (10)	C13—C12—H12B	109.4
O4—Zn2—O2E	101.9 (2)	C11—C12—H12B	109.4
O5—Zn2—O2E	133.7 (2)	H12A—C12—H12B	108.0
O2—Zn2—O2E	95.5 (2)	C14—C13—C12	111.1 (3)
O4—Zn2—O3B	125.86 (18)	C14—C13—H13A	109.4
O5—Zn2—O3B	89.12 (13)	C12—C13—H13A	109.4
O2—Zn2—O3B	115.6 (2)	C14—C13—H13B	109.4
O2E—Zn2—O3B	45.1 (2)	C12—C13—H13B	109.4
O4—Zn2—O2B	89.76 (16)	H13A—C13—H13B	108.0
O5—Zn2—O2B	143.75 (11)	C13—C14—C15	111.2 (3)
O2—Zn2—O2B	97.72 (18)	C13—C14—H14A	109.4
O2E—Zn2—O2B	12.2 (2)	C15—C14—H14A	109.4
O3B—Zn2—O2B	54.68 (10)	C13—C14—H14B	109.4
O4—Zn2—Zn1	85.10 (7)	C15—C14—H14B	109.4
O5—Zn2—Zn1	40.23 (6)	H14A—C14—H14B	108.0
O2—Zn2—Zn1	83.40 (8)	C14—C15—C10	114.3 (3)
O2E—Zn2—Zn1	172.9 (2)	C14—C15—H15A	108.7
O3B—Zn2—Zn1	129.32 (11)	C10—C15—H15A	108.7
O2B—Zn2—Zn1	174.83 (13)	C14—C15—H15B	108.7
C8—O2—Zn2	122.3 (2)	C10—C15—H15B	108.7
C18—N2—H2C	109.5	H15A—C15—H15B	107.6
C18—N2—H2D	109.5	C17—C16—C10	117.5 (3)
H2C—N2—H2D	109.5	C17—C16—H16A	107.9
C18—N2—H2E	109.5	C10—C16—H16A	107.9
H2C—N2—H2E	109.5	C17—C16—H16B	107.9
H2D—N2—H2E	109.5	C10—C16—H16B	107.9
C3—C2—C1	113.6 (3)	H16A—C16—H16B	107.2
C3—C2—H2A	108.8	O4—C17—O3	124.6 (3)
C1—C2—H2A	108.8	O4—C17—C16	116.2 (3)
C3—C2—H2B	108.8	O3—C17—C16	119.2 (3)
C1—C2—H2B	108.8	N2—C18—C10	114.6 (3)
H2A—C2—H2B	107.7	N2—C18—H18A	108.6
C17—O3—Zn1	136.7 (2)	C10—C18—H18A	108.6
C2—C3—C4	111.1 (4)	N2—C18—H18B	108.6
C2—C3—H3A	109.4	C10—C18—H18B	108.6
C4—C3—H3A	109.4	H18A—C18—H18B	107.6
C2—C3—H3B	109.4	O3A—N3A—O2A	117.1 (8)
C4—C3—H3B	109.4	O3A—N3A—O1A	117.3 (7)
H3A—C3—H3B	108.0	O2A—N3A—O1A	125.5 (7)
C17—O4—Zn2	123.8 (2)	O3D—N3D—O2D	112.5 (7)
C3—C4—C5	110.9 (3)	O3D—N3D—O1D	127.6 (7)
C3—C4—H4A	109.5	O2D—N3D—O1D	119.9 (7)
C5—C4—H4A	109.5	N5B—O2B—Zn2	86.3 (4)
C3—C4—H4B	109.5	N5B—O3B—Zn2	102.7 (4)
C5—C4—H4B	109.5	O1B—N5B—O3B	123.3 (6)
H4A—C4—H4B	108.1	O1B—N5B—O2B	120.0 (6)
Zn2—O5—Zn1^i^	127.54 (11)	O3B—N5B—O2B	115.9 (6)
Zn2—O5—Zn1	102.36 (10)	N5E—O2E—Zn2	112.5 (6)
Zn1^i^—O5—Zn1	96.72 (9)	O1E—N5E—O2E	122.7 (9)
Zn2—O5—H5C	98.0	O1E—N5E—O3E	119.7 (8)
Zn1^i^—O5—H5C	117.7	O2E—N5E—O3E	117.3 (8)
Zn1—O5—H5C	114.2	O3C—N4C—O1C	121.6 (9)
C6—C5—C4	111.6 (4)	O3C—N4C—O2C	120.0 (8)
C6—C5—H5A	109.3	O1C—N4C—O2C	118.4 (8)
C4—C5—H5A	109.3	O3F—N4F—O1F	125.7 (10)
C6—C5—H5B	109.3	O3F—N4F—O2F	118.7 (10)
C4—C5—H5B	109.3	O1F—N4F—O2F	112.4 (9)
			
O5—Zn1—O1—C8	9.3 (4)	O7—Zn1—O5—Zn2	−139.60 (11)
O6—Zn1—O1—C8	−169.7 (4)	O3—Zn1—O5—Zn2	44.86 (11)
O7—Zn1—O1—C8	102.8 (4)	Zn1^i^—Zn1—O5—Zn2	130.84 (13)
O3—Zn1—O1—C8	−86.1 (4)	O1—Zn1—O5—Zn1^i^	179.69 (10)
Zn1^i^—Zn1—O1—C8	9.6 (4)	O5^i^—Zn1—O5—Zn1^i^	0.0
Zn2—Zn1—O1—C8	−20.1 (3)	O7—Zn1—O5—Zn1^i^	89.57 (10)
O1—Zn1—Zn2—O4	−97.08 (12)	O3—Zn1—O5—Zn1^i^	−85.98 (10)
O5^i^—Zn1—Zn2—O4	84.68 (11)	Zn2—Zn1—O5—Zn1^i^	−130.84 (13)
O5—Zn1—Zn2—O4	136.68 (13)	C3—C4—C5—C6	55.1 (5)
O6—Zn1—Zn2—O4	−32.92 (16)	C4—C5—C6—C1	−55.2 (5)
O7—Zn1—Zn2—O4	−174.51 (12)	C9—C1—C6—C5	171.4 (3)
O3—Zn1—Zn2—O4	5.49 (11)	C2—C1—C6—C5	52.9 (4)
Zn1^i^—Zn1—Zn2—O4	104.53 (9)	C7—C1—C6—C5	−66.9 (4)
O1—Zn1—Zn2—O5	126.23 (13)	C9—C1—C7—C8	54.5 (4)
O5^i^—Zn1—Zn2—O5	−52.01 (14)	C2—C1—C7—C8	176.8 (3)
O6—Zn1—Zn2—O5	−169.60 (16)	C6—C1—C7—C8	−63.5 (4)
O7—Zn1—Zn2—O5	48.81 (13)	Zn2—O2—C8—O1	1.5 (5)
O3—Zn1—Zn2—O5	−131.19 (12)	Zn2—O2—C8—C7	−177.1 (2)
Zn1^i^—Zn1—Zn2—O5	−32.16 (10)	Zn1—O1—C8—O2	19.9 (6)
O1—Zn1—Zn2—O2	11.49 (12)	Zn1—O1—C8—C7	−161.4 (3)
O5^i^—Zn1—Zn2—O2	−166.75 (11)	C1—C7—C8—O2	101.5 (4)
O5—Zn1—Zn2—O2	−114.75 (13)	C1—C7—C8—O1	−77.2 (4)
O6—Zn1—Zn2—O2	75.65 (16)	C2—C1—C9—N1	−73.7 (4)
O7—Zn1—Zn2—O2	−65.94 (12)	C6—C1—C9—N1	169.0 (3)
O3—Zn1—Zn2—O2	114.06 (12)	C7—C1—C9—N1	47.9 (4)
Zn1^i^—Zn1—Zn2—O2	−146.91 (9)	C18—C10—C11—C12	−66.4 (4)
O1—Zn1—Zn2—O3B	129.3 (2)	C15—C10—C11—C12	52.2 (4)
O5^i^—Zn1—Zn2—O3B	−48.9 (2)	C16—C10—C11—C12	169.1 (3)
O5—Zn1—Zn2—O3B	3.1 (2)	C10—C11—C12—C13	−55.9 (4)
O6—Zn1—Zn2—O3B	−166.5 (3)	C11—C12—C13—C14	56.2 (5)
O7—Zn1—Zn2—O3B	51.9 (2)	C12—C13—C14—C15	−55.0 (5)
O3—Zn1—Zn2—O3B	−128.1 (2)	C13—C14—C15—C10	54.1 (5)
Zn1^i^—Zn1—Zn2—O3B	−29.1 (2)	C18—C10—C15—C14	70.5 (4)
O4—Zn2—O2—C8	72.0 (3)	C11—C10—C15—C14	−51.7 (4)
O5—Zn2—O2—C8	−47.3 (3)	C16—C10—C15—C14	−171.2 (3)
O2E—Zn2—O2—C8	176.3 (3)	C18—C10—C16—C17	−87.8 (4)
O3B—Zn2—O2—C8	−141.4 (3)	C11—C10—C16—C17	37.9 (4)
O2B—Zn2—O2—C8	164.2 (3)	C15—C10—C16—C17	156.4 (3)
Zn1—Zn2—O2—C8	−10.7 (3)	Zn2—O4—C17—O3	5.5 (5)
C9—C1—C2—C3	−169.1 (4)	Zn2—O4—C17—C16	−174.2 (2)
C6—C1—C2—C3	−53.6 (5)	Zn1—O3—C17—O4	4.3 (6)
C7—C1—C2—C3	67.5 (4)	Zn1—O3—C17—C16	−176.0 (2)
O1—Zn1—O3—C17	59.8 (3)	C10—C16—C17—O4	−122.7 (4)
O5^i^—Zn1—O3—C17	−117.4 (3)	C10—C16—C17—O3	57.6 (5)
O5—Zn1—O3—C17	−34.5 (3)	C11—C10—C18—N2	−54.5 (4)
O6—Zn1—O3—C17	152.2 (3)	C15—C10—C18—N2	−174.4 (3)
Zn1^i^—Zn1—O3—C17	−76.0 (3)	C16—C10—C18—N2	70.0 (4)
Zn2—Zn1—O3—C17	−7.2 (3)	O4—Zn2—O2B—N5B	−132.7 (5)
C1—C2—C3—C4	56.0 (5)	O5—Zn2—O2B—N5B	−0.2 (7)
O5—Zn2—O4—C17	21.5 (3)	O2—Zn2—O2B—N5B	119.4 (5)
O2—Zn2—O4—C17	−88.9 (3)	O2E—Zn2—O2B—N5B	39.1 (11)
O2E—Zn2—O4—C17	171.3 (3)	O3B—Zn2—O2B—N5B	3.5 (5)
O3B—Zn2—O4—C17	128.8 (3)	O4—Zn2—O3B—N5B	55.1 (6)
O2B—Zn2—O4—C17	173.0 (3)	O5—Zn2—O3B—N5B	174.2 (5)
Zn1—Zn2—O4—C17	−7.5 (3)	O2—Zn2—O3B—N5B	−84.6 (5)
C2—C3—C4—C5	−55.2 (5)	O2E—Zn2—O3B—N5B	−13.7 (5)
O4—Zn2—O5—Zn1^i^	60.31 (17)	O2B—Zn2—O3B—N5B	−3.6 (5)
O2—Zn2—O5—Zn1^i^	175.16 (14)	Zn1—Zn2—O3B—N5B	172.2 (4)
O2E—Zn2—O5—Zn1^i^	−76.7 (3)	Zn2—O3B—N5B—O1B	176.3 (7)
O3B—Zn2—O5—Zn1^i^	−69.0 (2)	Zn2—O3B—N5B—O2B	6.4 (9)
O2B—Zn2—O5—Zn1^i^	−66.0 (4)	Zn2—O2B—N5B—O1B	−175.7 (8)
Zn1—Zn2—O5—Zn1^i^	108.63 (16)	Zn2—O2B—N5B—O3B	−5.4 (8)
O4—Zn2—O5—Zn1	−48.32 (13)	O4—Zn2—O2E—N5E	−88.7 (7)
O2—Zn2—O5—Zn1	66.53 (12)	O5—Zn2—O2E—N5E	51.7 (7)
O2E—Zn2—O5—Zn1	174.6 (3)	O2—Zn2—O2E—N5E	161.8 (7)
O3B—Zn2—O5—Zn1	−177.62 (19)	O3B—Zn2—O2E—N5E	40.8 (6)
O2B—Zn2—O5—Zn1	−174.7 (3)	O2B—Zn2—O2E—N5E	−97.1 (14)
O1—Zn1—O5—Zn2	−49.48 (12)	Zn2—O2E—N5E—O1E	−172.9 (7)
O5^i^—Zn1—O5—Zn2	130.84 (13)	Zn2—O2E—N5E—O3E	0.5 (11)

Symmetry codes: (i) −*x*, −*y*+1, −*z*+1.

### Hydrogen-bond geometry (Å, °)

**Table:**

*D*—H···*A*	*D*—H	H···*A*	*D*···*A*	*D*—H···*A*
N1—H1C···O1	0.91	2.23	2.918 (4)	132
N1—H1D···O2C^ii^	0.91	2.18	3.003 (9)	149
N1—H1E···O1B^ii^	0.91	2.57	3.015 (6)	111
N1—H1E···O2D^iii^	0.91	2.25	3.090 (8)	153
N1—H1E···O3D^iii^	0.91	2.31	3.037 (8)	136
N2—H2C···O1D^iv^	0.91	2.00	2.906 (8)	172
N2—H2D···O1C^ii^	0.91	2.02	2.884 (9)	158
N2—H2E···O3	0.91	2.01	2.748 (5)	138
O5—H5C···O1D	0.95	1.86	2.809 (8)	173
O6—H6C···O2C^ii^	0.98	1.97	2.944 (7)	170
O6—H6C···O2C^i^	0.98	2.01	2.819 (7)	138
O6—H6D···O3D^iv^	0.95	2.58	3.010 (9)	108
O6—H6D···O3B^i^	0.95	2.00	2.927 (5)	163
O7—H7C···O3C^i^	0.95	1.78	2.735 (9)	178
O7—H7D···O2D	0.96	1.87	2.819 (8)	173

Symmetry codes:  (ii) *x*−1, *y*+1, *z*; (iii) −*x*, −*y*+2, −*z*+1; (iv) *x*−1, *y*, *z*; (i) −*x*, −*y*+1, −*z*+1.
